# Laparoscopic Finding of Ectopic Adrenocortical Tissue in a 2-Year-Old Boy with Vanishing Testis

**DOI:** 10.1055/s-0037-1612633

**Published:** 2018-01-10

**Authors:** Antonio Marte

**Affiliations:** 1 Department of Pediatric Surgery, Università della Campania Luigi Vanvitelli, Naples, Italy

**Keywords:** ectopic adrenocortical tissue, laparoscopy, vanishing testis

## Abstract

Ectopic adrenocortical tissue (EAT) along the spermatic cord is an unusual condition in children. The author reports on a 2-year-old boy with impalpable testis. On laparoscopy, EAT was detected along the hypotrophic spermatic vessels and excised. These remnants should be removed to prevent hormone production or malignant transformation.

## Introduction

Ectopic adrenocortical tissue (EAT) along the spermatic cord is an uncommon finding in children who undergo surgical groin exploration with an overall incidence of 1 to 9.3%. 1 The majority of patients are younger than 2 years of age and the occurrence seems significantly increased with undescended testes. 2 In a case study of 935 groin explorations, Sullivan et al reported a prevalence of 0.7% at inguinal herniotomy, 4.1% at ligation of the patent processus vaginalis for communicating hydrocele, and 3.3% at exploration for undescended testes. 3

The ectopic adrenal tissue appears as a small (1–5 mm) bright yellow soft nodule, clearly different in color and consistency from fat, and embedded between cremasteric fibers. With the progressive increase in laparoscopy for inguinal hernia repair and nonpalpable testes in children, as in open inguinal surgery. Pediatric surgeons should expect to find EAT during these procedures. The author reports on a case of EAT in a child who underwent laparoscopic exploration of a nonpalpable left testis at his institution.

## Case Report

**Fig. 1:**
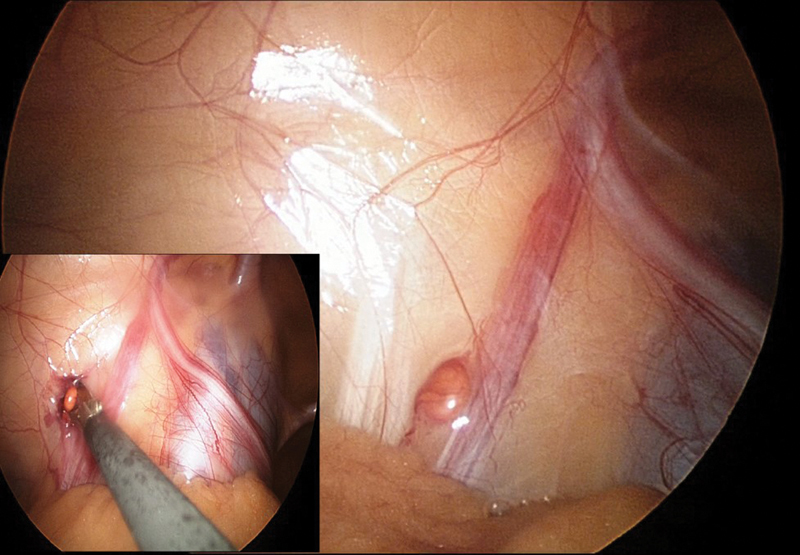
Laparoscopic appearance of ectopic adrenocortical tissue covered by peritoneum and its excision using a 3-mm operative trocar.

**Fig. 2:**
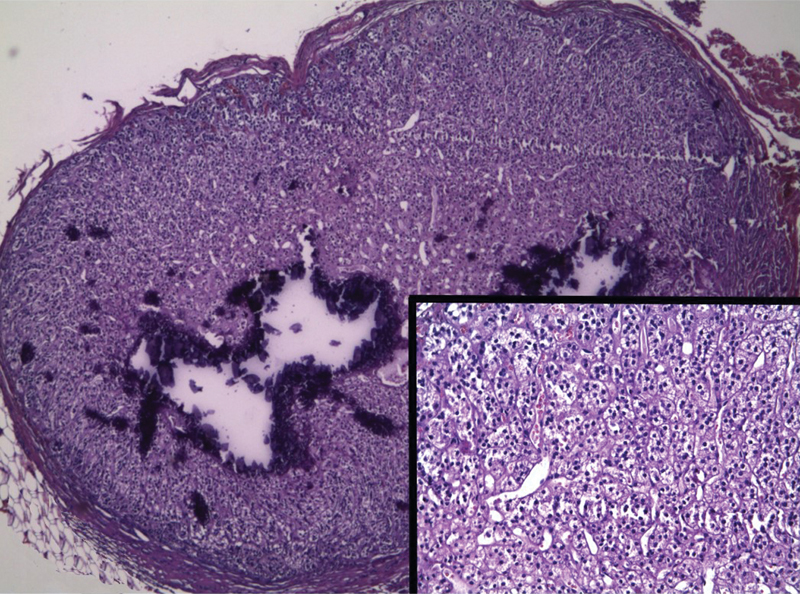
Histologic section of the excised nodule, showing encapsulated adrenal cortical tissue and focal calcifications (hematoxylin and eosin, HE, ×50).Inset: detail of ectopic adrenocortical tissue showing no medullary tissue or atypical cells (HE ×150).

A 2-year-old boy had neonatal diagnosis of a nonpalpable left testicle. After a delay due to uncertainty by his parents, the boy came to us for laparoscopic exploration. The patient underwent standard laparoscopy with a reusable transumbilical 5-mm port with a 0-degree optic. Laparoscopic exploration showed a closed left internal ring, hypotrophic vessels, and a vas deferens entering the internal inguinal ring. A bright yellow nodule of approximately 4 mm stood out over the vessels 3 cm from the inner inguinal ring ( Fig. 1 ). Suspecting the presence of EAT, the author decided to go ahead with the excision of the nodule. An additional contralateral right 3 mm trocar was introduced on the right side of the abdomen; a small window was created in the peritoneum, overlapping the nodule, and the nodule was easily isolated and excised electrocoagulating its thin vessels (not recommended in case of vital testis). Histopathological examination showed an oval-shaped nodule consisting of a small adrenal cortical nodule, with no medullary tissue or atypical cells and some microcalcifications ( Fig. 2 ). At the end of the laparoscopy, given that the internal ring was closed, the testicular nubbin was excised through a small inguinal incision. 4 The histological exam revealed fibrous–muscular–adipose tissue.

## Discussion

In 1740, Morgagni first described ectopic adrenal tissue in the vicinity of the adrenal gland, and in 1885, Dagonest observed the presence of adrenocortical tissue in the spermatic cord of an infant. 5 6 These remnants are usually found in the pediatric age but are also described in adults. 7 8 Ectopic adrenal cortical tissue can be found in the upper abdomen or anywhere along the track of the gonadal descent. The most frequent sites are celiac axis area (32%), broad ligament (23%), adnexa of the testes (7.5%), kidney (subcapsular upper pole) (0.1–6%), and spermatic cord (1–9.3%). 9 10 These anatomic locations can be explained on an embryologic basis given the close spatial relationship between the developing kidneys and adrenal glands. There are even unusual anatomic sites where one can find these EAT, such as placenta, liver, lung, and intracranial cavity. 11 General occurrence in various publications refers to patients who have experienced surgical groin explorations (1.2%). In a recent review, Mendez et al found EAT in 13 out of 1,120 surgical groin exploration (1.16%) including 6 inguinal hernias, 5 undescended testes, and 2 communicating hydroceles. Moreover, in their review of the literature until 2006, less than 100 cases were described near the genital structures. 1 Some cases of EAT have also been reported in girls, although the lower incidence in girls is not easy to explain. Oğuz et al, 12 in a multicenter study, reported an incidence of EAT of 2.02% out of a total of 296 male patients who had inguinoscrotal surgery between 2009 and 2011. Vaos et al 13 reported a comprehensive incidence of 2.5% of EAT in 316 inguinoscrotal operations performed on 289 consecutive patients (0.7% in girls and 1.8% in boys). In this report, the nodules were located along the spermatic cord, in the apex of the hernial sac, and between the testis and epididymis. Some authors have estimated that these remnants may be present in 50% of newborns but most of them become atrophic by adult life. 14 Other authors 2 suggest that there is a significant increased incidence of EAT in patients with undescended testis: out of 1,069 patients (159 girls and 910 boys), the incidence of EAT totaled 1.63% in the cases of hernia and hydrocele of cord, with 5.1% in cases of undescended testis. No EAT was found in girls. To our knowledge, this is the first published case of EAT found laparoscopically in a case of vanishing testis. Laparoscopic exploration for nonpalpable testis highlighted EAT along the hypotrophic spermatic vessels in their intra-abdominal course. Macroscopically, the appearance of ectopic adrenal tissue was characteristic: a round, yellow nodule, firm in consistency, standing out clearly on the floor of the peritoneum almost superimposed on the testicular vessels. This finding is quite different from what is usually found during groin surgery where the EAT, when present, appears firmly embedded in the cremasteric fibers that surround the spermatic elements, resembling a fat lobule. In fact, these remnants may undergo hyperplasia in conditions associated with excessive adrenocorticotropic hormone production and may occasionally give rise to neoplasms. 15 Although the occurrence of neoplasm in ectopic adrenal nodules is far from common, pheochromocytoma, Leydig cell tumor, and adrenal adenoma have been reported. 16 17 18 Moreover, primary neoplastic tissue in the spermatic cord is also rare but paragangliomas have been described. 19 In conclusion, on the basis of this observation, the author thinks it is important for the surgeon to expect the possibility of a nodule consistent with EAT during groin procedures and also laparoscopy for nonpalpable testis. These remnants should be removed to prevent hormone production or malignant transformation.

## New Insights and the Importance for the Pediatric Surgeon

This is the first report on a case of ectopic adrenocortical tissue found and treated laparoscopically in a case of vanishing testis in a child.

## References

[1] Mendez R Tellado M G Somoza I Ectopic adrenal tissue in the spermatic cord in pediatric patients: surgical implications Int Braz J Urol 2006 32 02 202 207, discussion 207 16650300 10.1590/s1677-55382006000200013

[2] Ozel S K Kazez A Akpolat N Presence of ectopic adrenocortical tissues in inguinoscrotal region suggests an association with undescended testis Pediatr Surg Int 2007 23 02 171 175 17066272 10.1007/s00383-006-1826-1

[3] Sullivan J G Gohel M Kinder R B Ectopic adrenocortical tissue found at groin exploration in children: incidence in relation to diagnosis, age and sex BJU Int 2005 95 03 407 410 15679804 10.1111/j.1464-410X.2005.05310.x

[4] Sturm R Kurzrock E Amend G Shannon R Gong E Cheng E Blind ending vessels on diagnostic laparoscopy for nonpalpable testis: Is a nubbin present? J Pediatr Urol 2017 13 04 3920 3.92E8 10.1016/j.jpurol.2017.04.010 28666917

[5] Schechter D C Aberrant adrenal tissue Ann Surg 1968 167 03 421 426 5638527 10.1097/00000658-196803000-00017 PMC1387073

[6] Okur H Küçükaydin M Kazez A Kontaş O Ectopic adrenal tissue in the inguinal region in children Pediatr Pathol Lab Med 1995 15 05 763 767 8597861 10.3109/15513819509027011

[7] Müllhaupt G Mordasini L Gramann T Ertel V Schmid H P Abt D Ectopic adrenocortical tissue in the spermatic cord in a 44-year-old man Urol Case Rep 2014 2 05 169 170 26958477 10.1016/j.eucr.2014.05.009 PMC4782098

[8] Anderson J R Ross A H Ectopic adrenal tissue in adults Postgrad Med J 1980 56 661 806 808 7267489 10.1136/pgmj.56.661.806 PMC2426068

[9] Lack E E Heterotopic and accessory adrenal tissues Washington, DC Armed Forces Institute of Pathology 1997 34 35

[10] Mares A J Shkolnik A Sacks M Feuchtwanger M M Aberrant (ectopic) adrenocortical tissue along the spermatic cord J Pediatr Surg 1980 15 03 289 292 6103925 10.1016/s0022-3468(80)80139-4

[11] Wiener M F Dallgaard S A Intracranial adrenal gland; a case report AMA Arch Pathol 1959 67 02 228 233 13616833

[12] Oğuz F Yildiz T Beytur A Evaluation of children with inguinoscrotal ectopic adrenal tissues Turk J Med Sci 2013 43 553 556. doi:10.3906/sag-1206-25

[13] Vaos G Zavras N Boukouvalea I Ectopic adrenocortical tissue along the inguinoscrotal path of children Int Surg 2006 91 03 125 128 16845852

[14] Czaplicki M Bablok L Kuzaka B Janczewski Z Heterotopic adrenal tissue Int Urol Nephrol 1985 17 02 177 181 4086234 10.1007/BF02082491

[15] Vela Navarrete R Barat A Berrocal A López de Alda A Quezada F Testicular adrenal rests tumor: a difficult diagnosis [in Spanish] Actas Urol Esp 1990 14 02 146 148 2378272

[16] O'Crowley C R Martland H S Adrenal heterotopia rests and so-called Grawitz tumors J Urol 1943 50 06 756 768

[17] Graham L S Celiac accessory adrenal gland Cancer 1953 6 01 149 152

[18] Abe T Matsuda H Shindo J Nonomura K Koyanagi T Ectopic pheochromocytoma arising in the spermatic cord 5 years after removal of bilateral carotid body tumors and adrenal pheochromocytomas Int J Urol 2000 7 03 110 111 10750890 10.1046/j.1442-2042.2000.00143.x

[19] Garaffa G Muneer A Freeman A Paraganglioma of the spermatic cord: case report and review of the literature Sci World J 2008 8 1256 1258 10.1100/tsw.2008.161 PMC5849074 19112537

